# Emerging trends and hotspots in intestinal microbiota research in sepsis: bibliometric analysis

**DOI:** 10.3389/fmed.2024.1510463

**Published:** 2024-11-13

**Authors:** Zhengyi Zhang, Meijie Yang, Tong Zhou, Yingjie Chen, Xiujuan Zhou, Kunlan Long

**Affiliations:** ^1^School of Clinical Medicine, Chengdu University of Traditional Chinese Medicine, Chengdu, China; ^2^Department of Critical Care Medicine, Hospital of Chengdu University of Traditional Chinese Medicine, Chengdu, China

**Keywords:** sepsis, intestinal microbiota, bibliometric, VOSviewer, CiteSpace

## Abstract

**Background:**

The association between the gut microbiota and sepsis has garnered attention in the field of intestinal research in sepsis. This study utilizes bibliometric methods to visualize and analyze the literature on gut microbiota research in sepsis from 2011 to 2024, providing a scientific foundation for research directions and key issues in this domain.

**Methods:**

Original articles and reviews of gut microbiota research in sepsis, which published in English between 2011 and 2024, were obtained from the Web of Science Core Collection on June 21, 2024. Python, VOSviewer, and CiteSpace software were used for the visual analysis of the retrieved data.

**Results:**

A total of 1,031 articles were analyzed, originating from 72 countries or regions, 1,614 research institutions, and 6,541 authors. The articles were published in 434 different journals, covering 89 different research fields. The number of publications and citations in this research area showed a significant growth trend from 2011 to 2024, with China, the United States, and the United Kingdom being the main research forces. Asada Leelahavanichkul from Thailand was identified as the most prolific author, making him the most authoritative expert in this field. “Nutrients” had the highest number of publications, while “Frontiers in Cellular and Infection Microbiology,” “Frontiers in Immunology” and “the International Journal of Molecular Sciences” have shown increasing attention to this field in the past 2 years. Author keywords appearing more than 100 times included “gut microbiota (GM),” “sepsis” and “microbiota.” Finally, this study identified “lipopolysaccharides (LPS),” “short-chain fatty acids (SCFAs),” “probiotics,” “fecal microbiota transplantation (FMT)” and “gut-liver axis” as the research hotspots and potential frontier directions in this field.

**Conclusion:**

This bibliometric study summarizes current important perspectives and offers comprehensive guidance between sepsis and intestinal microbiota, which may help researchers choose the most appropriate research directions.

## 1 Introduction

Sepsis is defined as a dysregulated response of the host to infection, leading to potentially life-threatening organ dysfunction (1). The progression of sepsis is both complex and rapid, often accompanied by a severe inflammatory response and multiple organ dysfunction syndrome, leading to significant pathological and physiological burdens (2). Sepsis and septic shock impact millions of individuals globally each year, with sepsis mortality rates at approximately 25% (often higher) (3), and septic shock mortality rates exceeding 40% (1). Despite considerable advancements in sepsis research, such as the early administration of antibiotics and supportive care since the inception of the “Surviving Sepsis Campaign” in 2002, treatment outcomes remain unsatisfactory, and mortality rates are still high (4–6). Hence, it is crucial to obtain a thorough comprehension of the pathophysiological processes of sepsis from various viewpoints and to investigate new therapies designed to lower mortality rates and enhance the long-term prognosis for sepsis patients.

Since the hypothesis that the gastrointestinal tract is the initiating organ of multiple organ dysfunction syndrome (MODS) was proposed in 1986 (7), numerous studies have substantiated the relationship between intestinal flora and sepsis (8–10). Emerging evidence indicates that microorganisms in the gut, such as eukaryotic viruses, bacteria, phages, and fungi, along with the metabolites they produce, are essential factors in determining susceptibility to sepsis and its outcomes (11). The significant impact of the gut microbiome on the host is mostly credited to the metabolites generated by beneficial gut bacteria, which play a crucial role in immune cell functioning (12). The immune response of the host is not only affected by metabolites derived from the gut microbiota but also by interactions among commensal bacteria that can control immune activation and change their metabolic response (11, 13). Abnormal gut microbiota can lead to digestive system symptoms such as diarrhea, dyspepsia, and constipation, as well as contribute to the development of non-gastrointestinal diseases like inflammatory bowel disease (IBD), non-alcoholic fatty liver disease (NAFLD), and sepsis (14–16). When gut microbiota is imbalanced, beneficial microbial populations decrease while harmful microbial populations increase, resulting in dysbiosis. Dysbiosis further promotes the proliferation of harmful microorganisms and disrupts the integrity of the intestinal barrier (9). Sepsis exacerbates this imbalance, leading to a reduction and collapse of normal intestinal microbiome diversity (10). The altered immune response during sepsis can modify the intestinal microbiome and induce inflammatory and oxidative stress pathways in the intestine, causing local dysbiosis. These changes significantly reduce beneficial anaerobes and compromise the integrity of the intestinal epithelium (17). Intestinal injury caused by sepsis allows microbes and endotoxins to translocate from the gut to extra-intestinal tissues, leading to tissue injury, organ dysfunction, and even death in sepsis patients (18, 19). Although the pathogenesis of sepsis is multifactorial and not fully understood, increasing evidence suggests that gut microbiome disorders predispose individuals to sepsis and adversely affect sepsis outcomes (20). As a result, utilizing gut microbiota for prognostic tools, therapeutic advancements, and targeted treatments could serve as successful approaches for managing sepsis. Having a thorough understanding of how gut microbiota impacts the development of sepsis could lead to the identification of potential microbiome markers that could be used for diagnosing, treating, and predicting the outcome of sepsis.

Given the growing body of literature on the correlation between sepsis and intestinal microbial communities, notably, no study has systematically summarized and analyzed, using bibliometric analysis, the association between intestinal microbial and sepsis. Consequently, the present study, by employing a detailed and systematic bibliometric analysis, seeks to explore the current landscape and emerging trends in “intestinal microbiota research in sepsis” from 2011 to 2024. Subjective overviews of relevant literature within specific fields are what earlier reviews offer; missing, however, is a thorough depiction of the collaboration and contributions among authors, countries, institutions, and journals. Furthermore, illustrating knowledge frameworks and identifying key research areas continue to be challenging, as evidenced by the paucity of systematic, comprehensive, and visual investigations in this domain. This study systematically examines new trends and hot spots in the field using bibliometric analysis. A benefit of bibliometrics as a tool is its ability to analyze research trends and focal points across various fields and sectors like management, sociology, economics, medicine, environmental engineering, and agriculture (21). Bibliometric techniques can help uncover current trends, popular topics, and interdisciplinary areas in scientific research. They also aid in assessing the impact and quality of academic accomplishments, ultimately offering valuable guidance for advancing scientific research (22). The objective of this research is to close this divide by employing bibliometric methods to evaluate quantitatively the studies on gut microbiota and sepsis. This will lay a strong groundwork for future research paths and address crucial issues in the field.

## 2 Materials and methods

### 2.1 Data source and retrieval

We conducted a thorough search of the Web of Science Core Collection (WoSCC) database from its inception to 2024. The search was finalized on June 21, 2024, resulting in the retrieval of 2,205 articles. The search strategy employed is detailed below:

1. “sepsis*” OR “septic shock*” OR “endotoxemia*” OR “SIRS” OR “systemic inflammatory response syndrome*”
2. “intestinal bacteria” OR “fecal bacteria” OR “gastrointestinal bacteria” OR “gut microbiota” OR “fecal microbiota” OR “gut flora” OR “gastrointestinal flora” OR “gastrointestinal microbiota” OR “gut microbiome” OR “intestinal microbiome” OR “fecal microbiome” OR “gut bacteria” OR “gastrointestinal microbiome” OR “intestinal flora” OR “gut microflora” OR “intestinal microflora” OR “fecal microflora” OR “gastrointestinal microflora” OR “intestinal microbiota” OR “fecal flora”
3. #1 AND #2

Two reviewers discovered potential discrepancies in the data search separately and then deliberated on them, ultimately reaching a consensus. We obtained a total of 2,057 articles by limiting the publication types to reviews and original articles and excluding articles in languages other than English.

### 2.2 Literature screening

**Figure 1:**
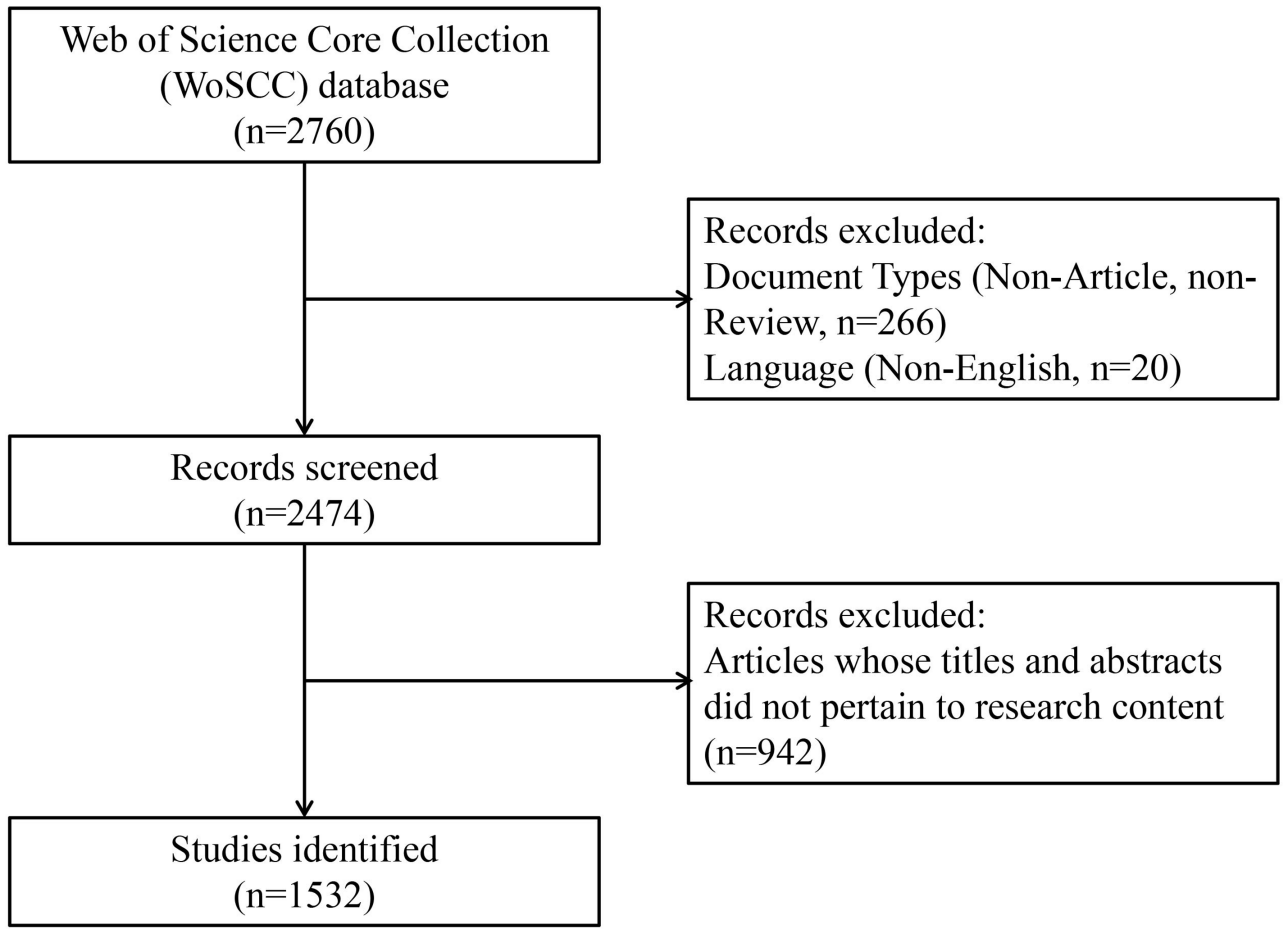
Flowchart of literature search selection.

Subsequently, we saved these articles in the “Marked List” of our Web of Science personal account for future analysis and screening. After excluding articles with titles and abstracts that did not align with the research content, we found 1,031 articles that satisfied the criteria for being included in the analysis (Figure 1).

Finally, we exported all the articles that satisfied the requirements in a “Plain Text File” format from the “Marked List.” This included the “Full Record and Cited References” for the subsequent stages of data cleansing and bibliometric analysis. In the “Plain Text File,” the information for each article is distributed across different field label lines. For example, the PT field tag indicates the start of an article’s information, while the ER field tag indicates its end. The full names of authors, keywords, all authors’ correspondence addresses (This includes countries and institutions that are connected or associated). The number of citations, the publication year, and the research areas are stored in the AF, DE, C1, TC, PY, and WC field tags, respectively.

### 2.3 Data cleaning

1. Keyword synonym replacement: In order to prevent loss of information caused by synonymy in keywords, we conducted deduplication and replaced synonyms in the keywords. For instance, “AKI” and “acute kidney injury” were unified as “acute kidney injury (AKI).”
2. Verification of author identities: We carried out comprehensive checks to confirm the identities of authors who shablue similar or identical names in order to avoid any confusion. In addition to utilizing ORCID data for confirming identities, we also cross-referenced information with trustworthy sources like official institutional websites and encyclopedias.
3. Standardization of Chinese author name spelling: We standardized the spelling of Chinese author names, such as Zhang Danying to Zhang Dan-Ying, and Zhang Lidi to Zhang Li-Di.

These processes guarantee the precision and uniformity of our data, which are essential for maintaining the integrity and dependability of our bibliometric analysis.

### 2.4 Bibliometric analysis

All raw data were extracted from the WoSCC database, and the following bibliometric analysis was carried out using three software applications: CiteSpace, VOSviewer, and Python.

1. The Web of Science (WoS) Core Collection database is a comprehensive academic literature database that covers a wide range of academic fields, including science, technology, medicine, and social sciences. It includes journal articles, conference papers, and other academic documents. Recognized as one of the authoritative academic literature resources, the WoS database holds a significant position in the academic community (23). Hence, our study opted for the WoS database.
2. CiteSpace is a software tool designed to visualize and analyze citation networks in academic literature. We used CiteSpace to conduct burst detection analysis on references and author keywords, enabling us to identify important references and research topics that saw a surge in influence within specific time periods. Cluster analysis of references provided insights into influential papers within academic fields (24).
3. We used VOSviewer 1.6.19 for visualizing and analyzing authors, institutions, countries and author keywords. In the generated network map, each node represents different countries, institutions, authors, or author keywords. The number of publications is illustrated by the size of the nodes, while varying colors correspond to distinct clusters or years. The lines connecting the nodes expose collaborative or citation relationships, with thicker lines indicating closer relationships or more citations. This tool has been widely used in the field of bibliometrics, as evidenced by articles previously published by the Ma et al. (25).
4. Python is a sophisticated programming language that is extensively utilized in various fields such as web development, data science, artificial intelligence, and more. We used Python to extract various important characteristics from the literature and compile bibliometric data tables for countries, institutions, authors, journals, and research areas (26). The analysis involved tallying the overall count of publications, *H*-index and total number of citations, which is a metric used to assess academic accomplishments and gauge the academic output and influence of scholars (27). In order to make the data easier to understand, we created bubble charts that illustrate the yearly publication patterns of journals, research categories, and author keywords. Within these bubble charts, the relative significance of a specific journal, research field, or author keyword in a given year is represented by the size of each bubble, with the number inside indicating the corresponding number of publications for that year (26).

## 3 Results

### 3.1 The annual trend of paper publication quantity and citation times

**Figure 2:**
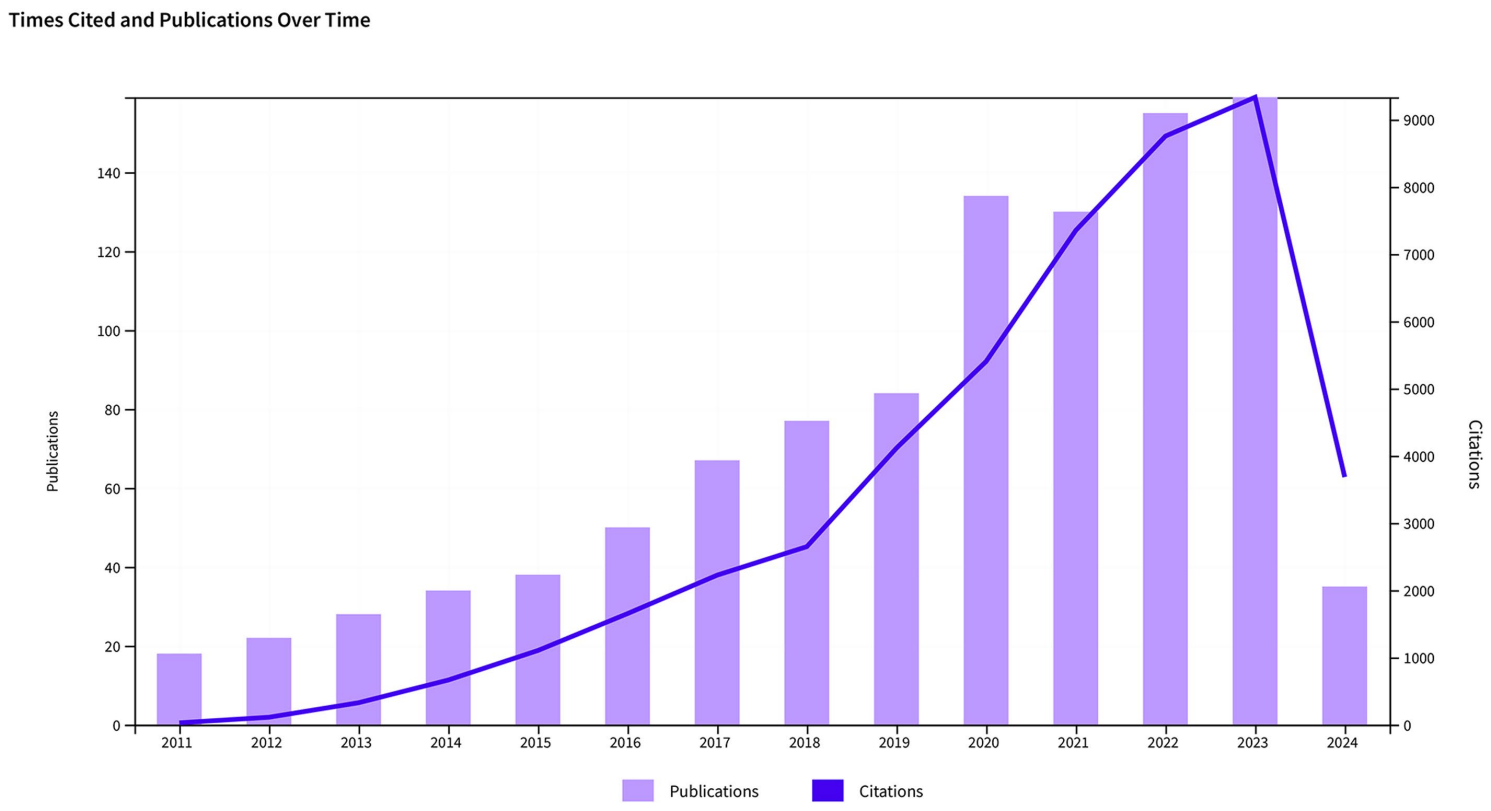
Trends in the growth of publications and the number of citations in sepsis and intestinal microbiota.

Among the 1,031 articles included in the analysis, 745 were original research articles, representing 72.26% of the total, and 286 were review articles, accounting for 27.74%. In Figure 2, the annual trends for publication numbers and citation frequencies are displayed. From 2011 to 2024, both the number of publications and citation times in this research field have shown an upward trend. Specifically, the largest increase in the number of articles occurred from 2019 to 2020, rising from 84 to 134. In 2023, the total number of publications reached 159, nearly nine times the number in 2011, which was 18 articles. This indicates that research activity in this field is continuously increasing. The substantial increase in citation frequency also highlights the growing influence and recognition of research in this field. Since 2018, there has been a significant rise in citation times, particularly from 2020 to 2021, with an increase of nearly 2,000 citations in just 1 year. It is important to mention that although only a quarter of 2024 has passed, publications in this particular area have garnered 3,079 citations, demonstrating a sustained growth in the influence of forthcoming research concerning sepsis and gut microbiota.

### 3.2 Analysis of authors

In Table 1, we can see the top 10 core authors along with their publication count, total citations, and *H*-index. Over the course of the database’s existence, a total of 6,541 authors have been involved in studies related to sepsis and intestinal microbiota, leading to the publication of 1,031 articles. Overall, most authors have not published many papers. Among them, 5,615 authors have only published one paper, while only 68 have published five or more papers. In terms of publication quantity, the top 10 authors have published a total of 95 articles. Leelahavanichkul, Asada leads with 15 articles, followed by Cani, Patrice D. with 10 articles. In terms of total citations, Cani, Patrice D. leads with 4,684 citations, followed by Gillevet, Patrick M. and Bajaj, Jasmohan S. with 1,754 and 1,256 citations, respectively. Although these two authors have published fewer papers than the other eight, they rank among the top three in terms of citations. Leelahavanichkul, Asada and Cani, Patrice D. are also leading regarding the *H*-index. When evaluating prolific writers, it’s crucial to take into account not only the number and caliber of their articles but also the timing of their publications. The top authors hail from different countries or regions such as the UK, the US, and Mexico, among others. Notably, two authors from Mexico belong to the same institution.

**Table 1:** Contribution of the top 10 authors in sepsis and intestinal microbiota.

Rank	Author	Institution	Country	Publications	Citations	*H*-index
1	Leelahavanichkul, Asada	Chulalongkorn Univ	Thailand	15	327	11
2	Cani, Patrice D.	Catholic Univ Louvain	Belgium	10	4,684	10
3	Wiersinga, W. Joost	Univ Amsterdam	Netherlands	9	727	9
3	Embleton, Nicholas D.	Royal Victoria Infirm	United Kingdom	9	639	9
3	Berrington, Janet Elizabeth	Newcastle Hosp NHS Fdn Trust	United Kingdom	9	639	9
3	Gasbarrini, Antonio	Agostino Gemelli Hosp	Italy	9	441	4
3	Tovar, Armando R.	Inst Nacl Ciencias Med & Nutr Salvador Zubiran	Mexico	9	339	7
3	Torres, Nimbe	Inst Nacl Ciencias Med & Nutr Salvador Zubiran	Mexico	9	339	7
9	Gillevet, Patrick M.	George Mason Univ	United States	8	1,754	8
9	Bajaj, Jasmohan S.	Virginia Commonwealth Univ	United States	8	1,256	8

**Figure 3:**
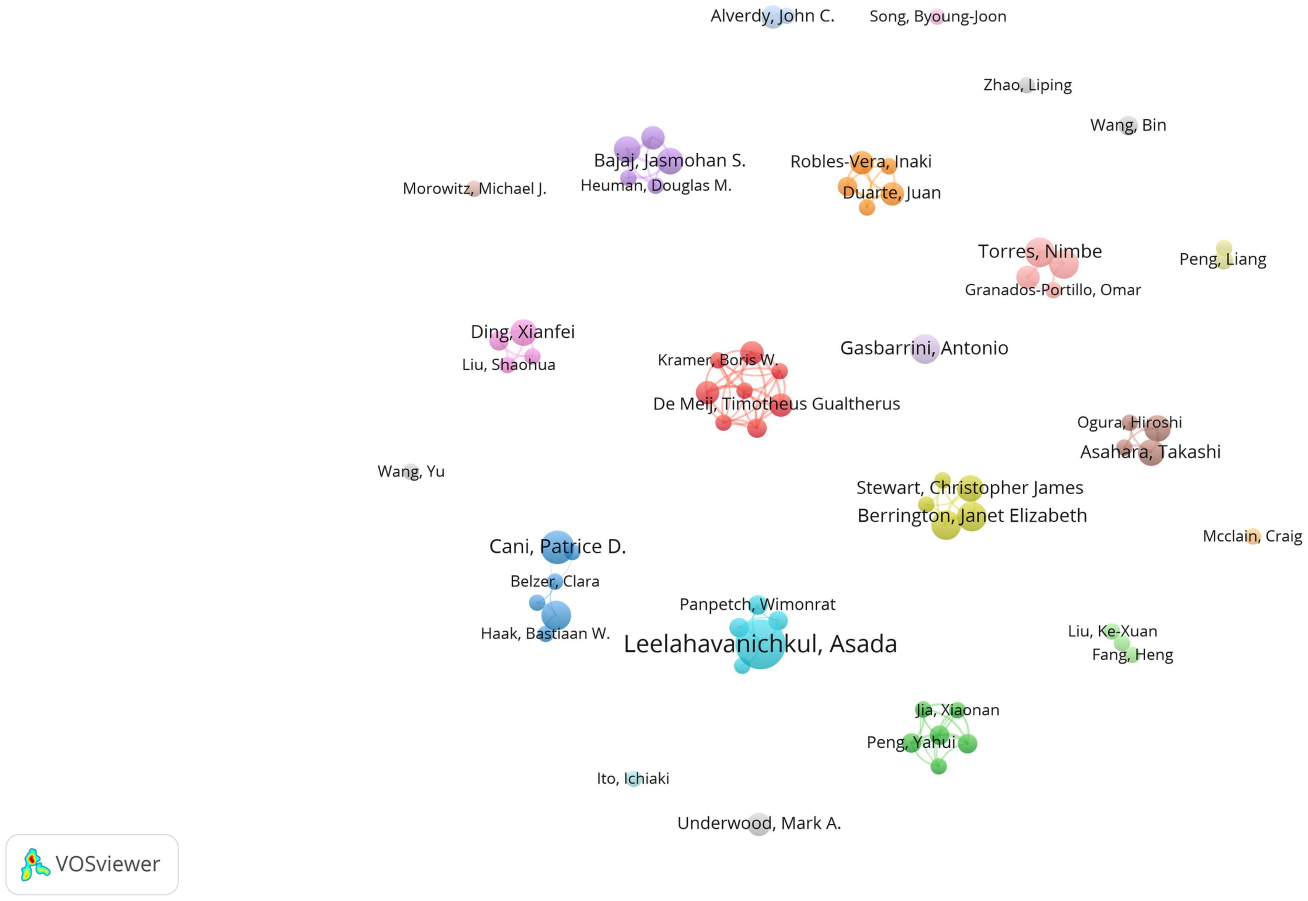
Cooperation map of authors in sepsis and intestinal microbiota.

Figure 3 illustrates the collaboration among authors in this field using VOSviewer software. In our statistical analysis, a requirement was established for authors to have a minimum of five published papers, resulting in only 68 authors meeting this threshold in the end. We also attempted to analyze the network graph after removing unconnected nodes. However, the result showed that the network graph only included eight authors. Therefore, we ultimately did not remove them, indicating a relatively loose connection among the authors. The nodes in the network visualization are sized according to the authors’ contribution levels, with larger nodes representing authors who have made a higher number of contributions in terms of published articles.

### 3.3 Analysis of institutions

A grand total of 1,614 institutions have consistently released articles on sepsis and the intestinal microbiome. Among the top 10 institutions by publication volume, half are from China and three are from the United States. In terms of publications, Southern Medical University in China is ranked first with 23 publications, 403 total citations, and an *H*-index of 11. Following closely is Zhejiang University in China, which comes in second place with 20 publications, 561 total citations, and an *H*-index of 14. The University of Amsterdam in the Netherlands (17 publications, 1,244 citations) and the University of Chicago in the United States (17 publications, 881 citations) are tied for third place (Table 2).

**Table 2:** Contribution of the top 10 institutions in sepsis and intestinal microbiota.

Rank	Institution	Publications	Citations	*H*-index	Country
1	Southern Med Univ	23	403	11	China
2	Zhejiang Univ	20	561	14	China
3	Univ Amsterdam	17	1,244	12	Netherlands
3	Univ Chicago	17	881	13	United States
5	Shanghai Jiao Tong Univ	15	1,473	9	China
5	Univ Calif Davis	15	1,090	13	United States
5	Chulalongkorn Univ	15	327	11	Thailand
5	Harbin Med Univ	15	96	5	China
9	Zhengzhou Univ	14	193	7	China
10	Univ Florida	12	494	8	United States

**Figure 4:**
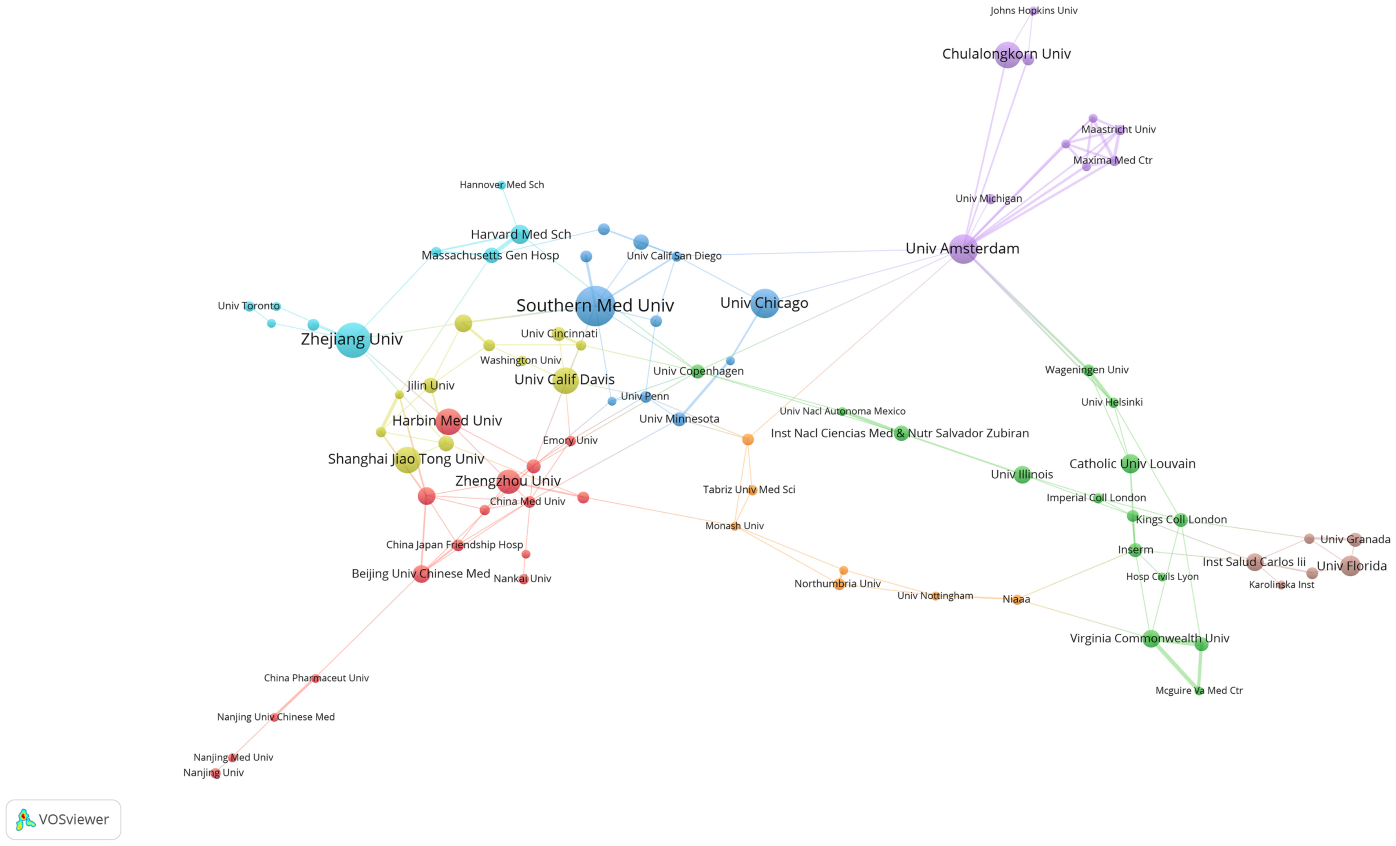
Cooperation map of institutions in sepsis and intestinal microbiota.

We utilized the VOSviewer software to perform an analysis on institutional collaboration, resulting in the development of an institutional collaboration network diagram (see Figure 4). To ensure a significant level of collaboration, we set the minimum publication threshold to five, leading to 100 institutions meeting this requirement. Subsequent removal of unconnected nodes left 85 institutions interconnected, signifying a strong level of collaboration among them.

### 3.4 Analysis of countries/regions

This collection of 1,031 articles originates from 72 countries/regions. Table 3 displays the top 10 countries/regions based on the total number of publications by all authors. China leads with the highest publication output, accounting for 34.72% of the total, and the United States comes next with 25.22%, followed by the United Kingdom with 6.21%. China and the United States stand out as the only countries with more than one hundblue published articles each. Even though China has a greater number of publications compablue to the United States, the latter leads in total citations and *H*-index, showcasing the superior quality of its articles.

**Table 3:** Contribution of the top 10 countries/regions in sepsis and intestinal microbiota.

Rank	Country	Publications	Citations	*H*-index	Average citations per publication	Number of cooperative countries	Multinational publications	Share of multinational cooperation publications
1	China	358	11,181	53	31.23	20	68	18.99
2	United States	260	15,627	65	60.1	40	114	43.85
3	United Kingdom	64	3,117	31	48.7	37	39	60.94
4	Italy	54	2,651	25	49.09	17	19	35.19
5	Japan	48	2,794	28	58.21	9	16	33.33
6	Spain	43	2,330	26	54.19	10	16	37.21
6	Canada	43	1,453	20	33.79	17	25	58.14
8	France	36	1,448	21	40.22	21	19	52.78
8	Germany	36	871	18	24.19	15	17	47.22
10	Netherlands	34	5,433	22	159.79	12	23	67.65

**Figure 5:**
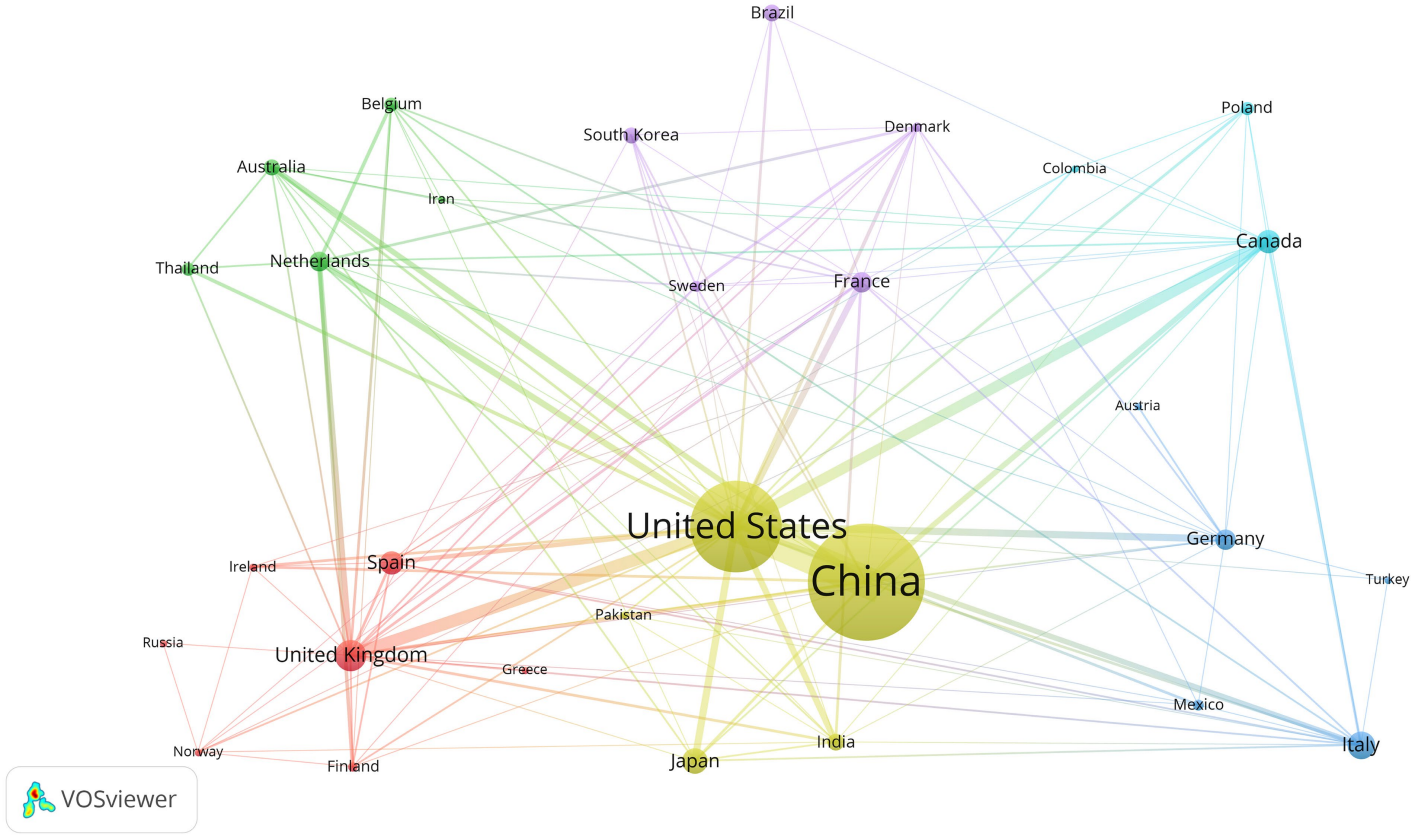
Cooperation map of countries regions in sepsis and intestinal microbiota.

We used VOSviewer software to analyze country/region data and created a network map to visualize collaborations between countries/regions (Figure 5). A minimum publication requirement of five was established, and 30 countries have met this requirement. The United States stands out as the most important global partner for other nations in this particular area.

### 3.5 Analysis of journals

Four hundred thirty-four different journals published a total of 1,031 articles on sepsis and gut microbiota. The top 10 journals contributing to this field are shown in Table 4. Nutrients leads in the number of publications with 36 articles (3.49%), closely trailed by Frontiers in Immunology with 29 articles (2.81%), PLoS One with 28 articles (2.71%), Scientific Reports with 27 articles (2.62%), and Frontiers in Microbiology with 22 articles (2.13%). PLoS One has the highest total number of citations, with 2,446 citations. Despite having only 14 articles published in this field, Gut Microbes holds a top position in total citations with 1,114 citations. This could be attributed to the high impact factor (IF) and broad coverage of the journal. According to the latest 2024 Journal Citation Reports (JCR) (28), Gut Microbes ranks highest in impact factor among the top 10 journals. By analyzing the average citation per publication (ACPP) of each journal, we observe that the majority of articles in this field have an ACPP higher than their impact factor (IF). This indicates that research on sepsis and gut microbiota receives a high level of citations, further demonstrating the strong academic interest of scholars in this field.

**Table 4:** Contribution of the top 10 journals in sepsis and intestinal microbiota.

Rank	Journal	Publications	Citations	Average citations per publication	The percentage of articles of institutions in total publications	IF
1	Nutrients	36	1,630	45.28	3.64	5.9
2	Frontiers in Immunology	29	796	27.45	2.94	7.3
3	PLoS One	28	2,446	87.36	2.83	3.7
4	Scientific Reports	27	1,974	73.11	2.73	4.6
5	Frontiers in Microbiology	22	488	22.18	2.23	5.2
6	International Journal of Molecular Sciences	20	540	27	2.02	5.6
6	Frontiers in Cellular and Infection Microbiology	20	215	10.75	2.02	5.7
8	Food & Function	15	296	19.73	1.52	6.1
9	Gut Microbes	14	1,114	79.57	1.42	12.2
9	Shock	14	277	19.79	1.42	3.1

**Figure 6:**
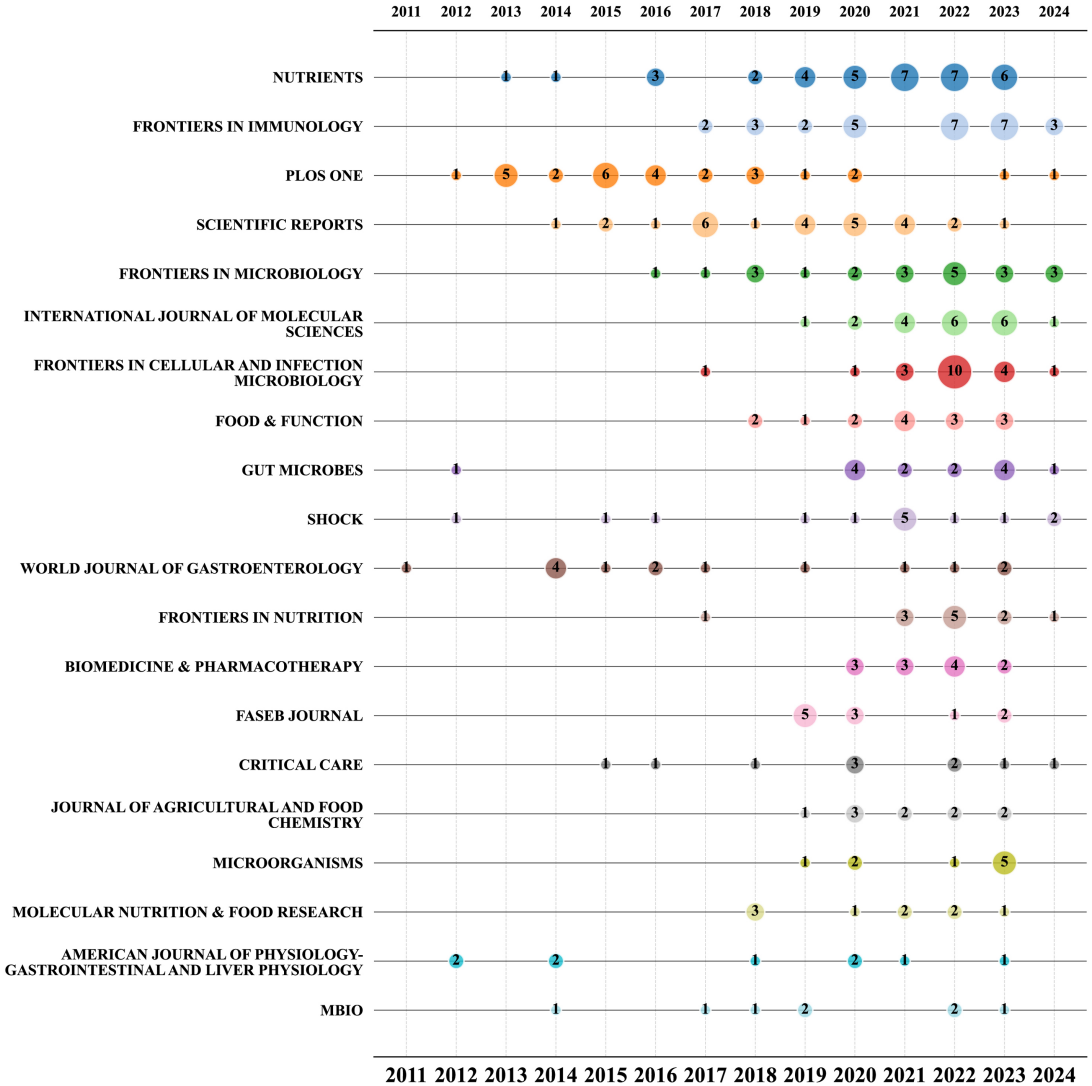
Bubble chart of the top 20 journals by year.

The bubble chart displayed in Figure 6 shows the top 20 journals based on the number of publications. The chart demonstrates that starting from 2021, *Nutrients, Frontiers in Cellular and Infection Microbiology, Frontiers in Immunology, and the International Journal of Molecular Sciences* have consistently maintained their position as the most prolific journals in this particular research field. Among them, Nutrients has maintained a relatively stable publication volume. Although PLoS One and Scientific Reports rank in the top 10 by total publication volume, their overall publication rates are declining, particularly notable with *PLoS One*, which had no articles published in this field in 2021 and 2022. Conversely, the publication volume of journals like *Frontiers in Cellular and Infection Microbiology* saw a significant surge in 2022, with the publication volume in this field more than tripling compared to the previous year.

### 3.6 Analysis of research fields

Studies examining the connection between sepsis and intestinal microbiota cover a wide range of 89 research fields. Table 5 displays the top 20 fields based on the volume of publications. The field of “Microbiology” boasts the highest publication volume, with 163 articles, followed by “Immunology” with 119 articles and “Nutrition & Dietetics” with 118 articles. In terms of total citation count, “Multidisciplinary Sciences,” “Gastroenterology & Hepatology,” and “Microbiology” are the leading fields, with 8,397, 7,288, and 6,635 citations, respectively. The category of “Multidisciplinary Sciences” has the highest average number of citations, with an average of 125.33 citations per paper. Despite having a limited number of 67 published articles, it has managed to attract significant attention and citations, showcasing the excellent quality of its research output. Conversely, the fields leading in publication volume lack high average citations per publication (ACPP), indicating that despite their extensive research output, they do not have a significant citation impact per article.

**Table 5:** Contribution of the top 20 research fields in sepsis and intestinal microbiota.

Rank	Research field	Publications	Citations	*H*-index	Average citations per publication	The percentage of articles of institutions in total publications
1	Microbiology	163	6,635	39	40.71	16.5
2	Immunology	119	3,256	30	27.36	12.04
3	Nutrition & Dietetics	118	4,998	42	42.36	11.94
4	Biochemistry & Molecular Biology	106	4,045	33	38.16	10.73
5	Gastroenterology & Hepatology	101	7,288	46	72.16	10.22
6	Medicine, Research & Experimental	78	2,663	26	34.14	7.89
7	Pharmacology & Pharmacy	72	1,863	21	25.88	7.29
8	Food Science & Technology	68	1,495	24	21.99	6.88
9	Multidisciplinary Sciences	67	8,397	37	125.33	6.78
10	Endocrinology & Metabolism	50	2,856	28	57.12	5.06
11	Pediatrics	47	1,784	20	37.96	4.76
12	Infectious Diseases	44	1,189	20	27.02	4.45
13	Cell Biology	43	2,002	22	46.56	4.35
14	Medicine, General & Internal	40	1,446	11	36.15	4.05
15	Surgery	39	988	19	25.33	3.95
16	Critical Care Medicine	34	1,372	17	40.35	3.44
17	Neurosciences	28	905	13	32.32	2.83
18	Chemistry, Multidisciplinary	25	609	11	24.36	2.53
18	Peripheral Vascular Disease	25	1,363	12	54.52	2.53
20	Chemistry, Applied	21	709	12	33.76	2.13

**Figure 7:**
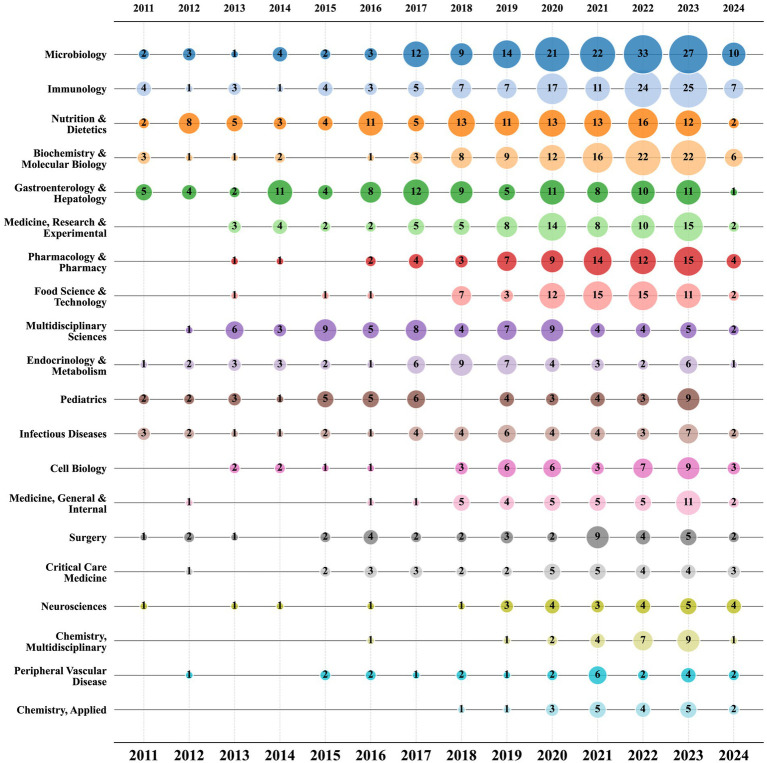
Bubble chart of the top 20 research areas by year.

In Figure 7, there is a bubble chart displaying the top 20 research fields based on the volume of publications. According to the chart, “Microbiology,” “Immunology,” and “Nutrition & Dietetics” have been consistently prominent research fields since 2011, underscoring their significant importance in the study of sepsis and gut microbiota. Since 2020, there has been a notable increase in publication volume in some fields, exhibiting a marked upward trend compared to previous years. Fields such as “Pharmacology & Pharmacy,” “Food Science & Technology,” and “Chemistry, Multidisciplinary” have shown significant growth. This analysis highlights the diversity and dynamics of the research fields related to sepsis and gut microbiota, showcasing both the broad interest in general fields and the significant impact of specialized fields. It also indicates the constant evolution of these areas, reflecting the expanding scope and depth of research in this domain.

### 3.7 Analysis of author keywords

**Figure 8:**
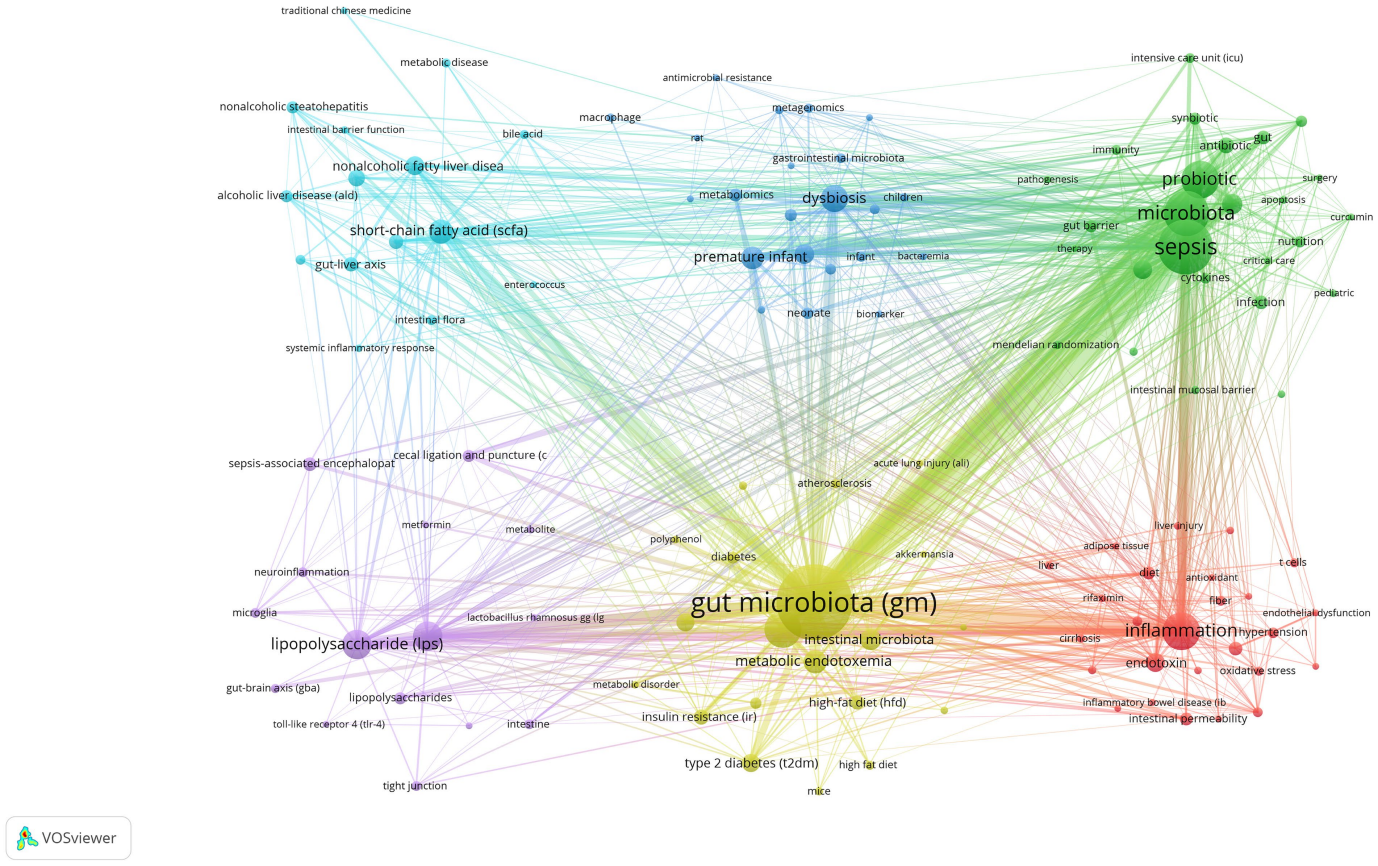
Cooperation map of author keywords in sepsis and intestinal microbiota.

After performing synonym substitution on key terms from various authors, we ultimately distilled 1,722 unique author keywords for analysis. Among these, 1,597 keywords appeared only 1 to 4 times, accounting for 92.74%. In contrast, 54 keywords appeared 10 times or more, accounting for 3.14%. The top 15 most frequently used keywords each appeared more than 30 times. Among them, “gut microbiota (GM),” “sepsis,” and “microbiota” ranked in the top three with 331, 185, and 132 occurrences, respectively (Table 6). Subsequently, we employed VOSviewer for further analysis to explore the relationships between keywords. By setting the minimum number of publications to 5, 124 author keywords met this threshold, allowing us to construct a keyword network graph (Figure 8).

**Table 6:** Contribution of the top 15 author keywords in sepsis and intestinal microbiota.

Rank	Author keywords	Total publications
1	Gut microbiota (GM)	331
2	Sepsis	185
3	Microbiota	132
4	Inflammation	97
5	Probiotic	96
6	Obesity	95
7	Lipopolysaccharide (LPS)	64
8	Endotoxemia	62
9	Dysbiosis	56
10	Short-chain fatty acid (SCFA)	44
11	Metabolic endotoxemia	42
12	Premature infant	40
13	Intestinal microbiota	35
14	Prebiotic	33
15	Necrotizing enterocolitis (NEC)	31

**Figure 9:**
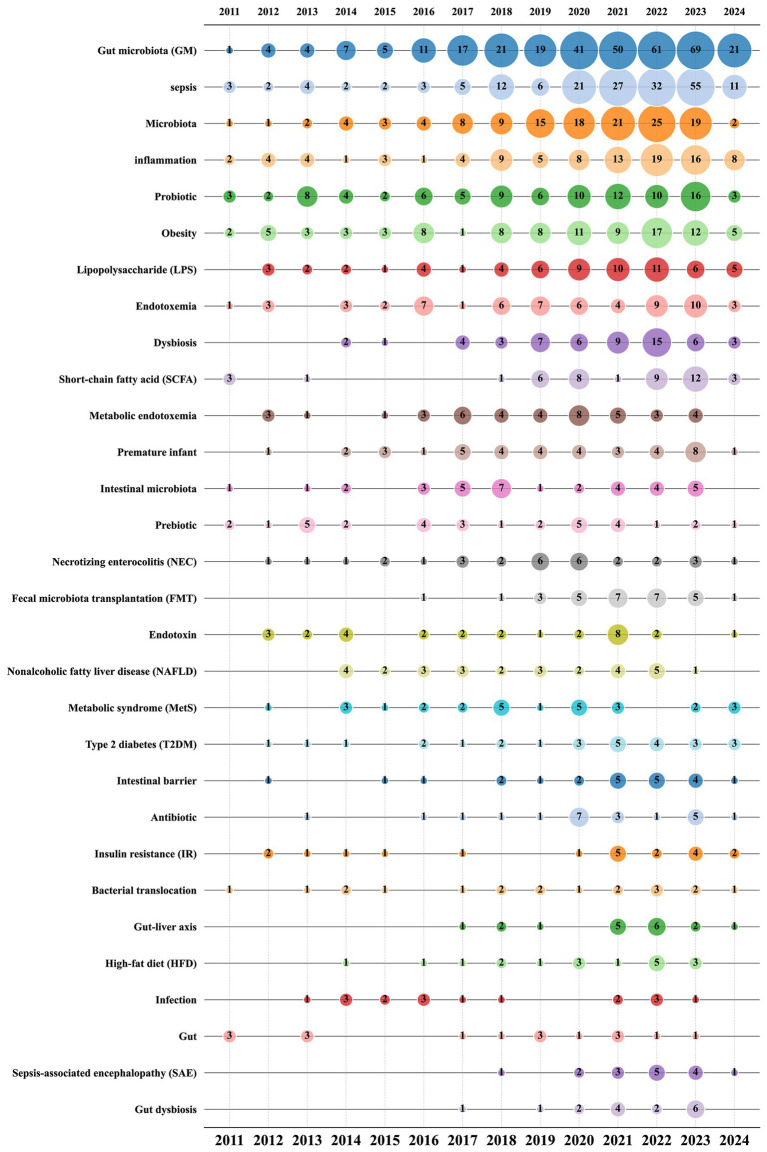
Bubble chart of the top 30 author keywords by year.

Figure 9 illustrates the total occurrences of the top 30 author keywords across different publication years, reflecting the dynamic trends in keyword usage over time. The keywords “gut microbiota (GM),” “sepsis,” and “microbiota” are the most prevalent, consistently ranking at the top and exhibiting a general trend of increasing occurrences year by year. This underscores their central role in research within this field. A detailed analysis of bubble sizes reveals that keywords like “metabolic endotoxemia,” “prebiotic,” “necrotizing enterocolitis (NEC),” and “endotoxin” have shown a decreasing trend over the past 5 years. This may indicate a shift in the research focus within the field, highlighting emerging areas of interest and evolving priorities in sepsis and gut microbiota studies.

**Figure 10:**
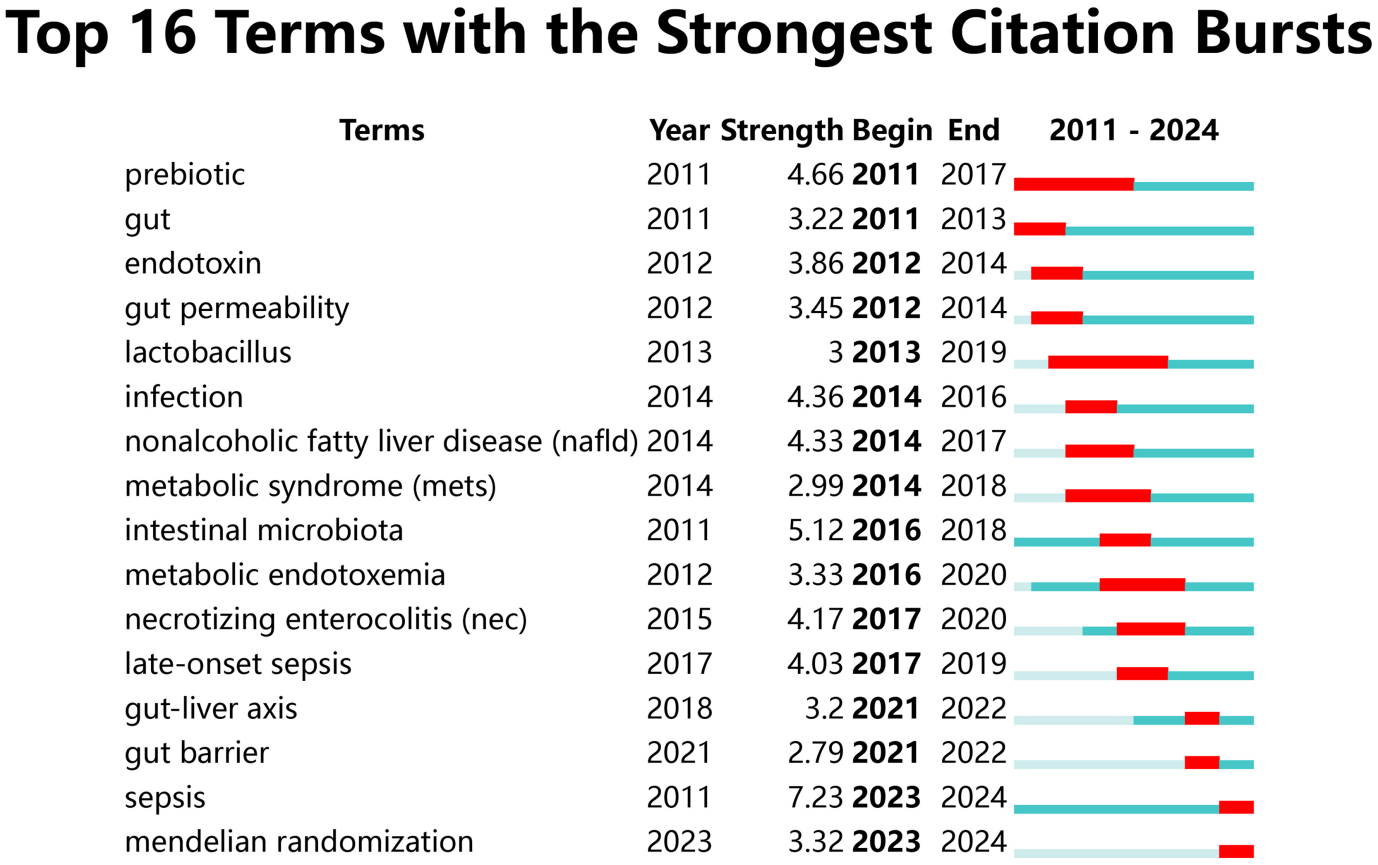
The clustered network map of Keywords in sepsis and intestinal microbiota.

In order to accurately identify keywords that may significantly influence the gut microbiota research in sepsis in recent years, we leveraged CiteSpace software to conduct a citation burst analysis on 1,031 articles. The analysis time frame spanned from 2011 to 2024, with the “Minimum Duration” parameter set to 2 years. The final results highlighted 16 key terms exhibiting the strongest citation bursts (Figure 10), where the gray lines denote time intervals and the red lines indicate burst duration. Among them, “sepsis” had the highest burst strength (7.23), followed by “intestinal microbiota” (5.12), “prebiotic “(4.66) and “gut-liver axis “(3.2). Notably, as of 2024, four keywords have emerged prominently: “gut-liver axis,” “gut barrier,” “sepsis,” and “Mendelian randomization.” Particularly, “sepsis” has remained highly prominent since its appearance in 2011, with a strength of 7.23. Although not among the top 30 most published topics, the majority of research on “gut barrier” and “Mendelian randomization” has occurred between 2021 and 2024, indicating that they have become recent focal points in this field. These findings suggest new directions and opportunities for future research in sepsis and gut microbiota-related studies.

From the citation burst analysis of the keywords, these keywords encompass the research hotspots in the “between intestinal microbiota and sepsis” field, which echoes the data in the bubble chart. Although the total number of publications on “lipopolysaccharides (LPS),” “Fecal microbiota transplantation (FMT),”and “short chain fatty acid (SCFA)” did not rank in the top 16, the majority of the relevant research articles were published from 2021 to 2024. This observation indicates that these keywords have unquestionably emerged as prominent areas of focus within the “intestinal research in sepsis” field over the past 2 years.

### 3.8 Analysis of reference

**Figure 11:**
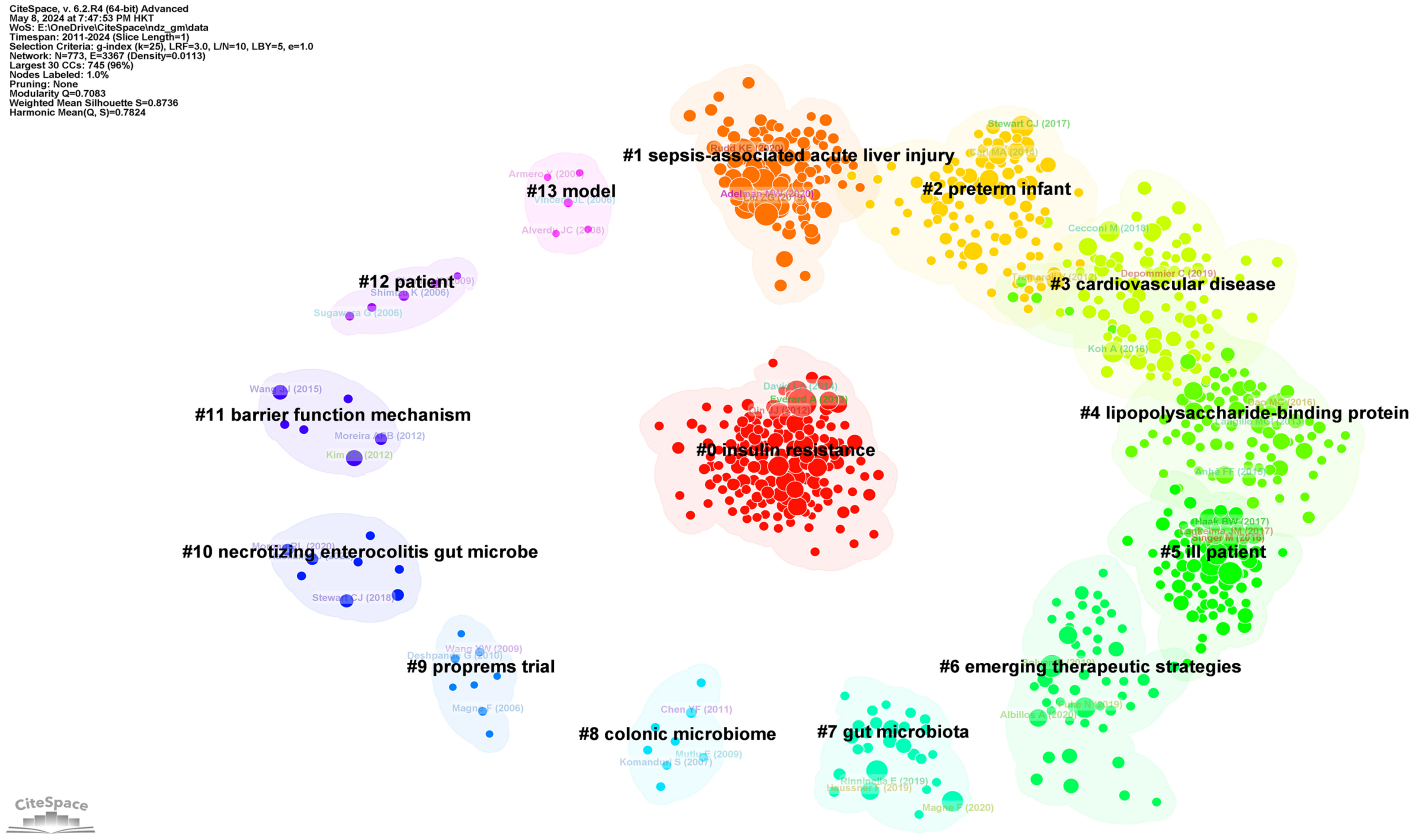
The clustered network map of reference in sepsis and intestinal microbiota.

**Figure 12:**
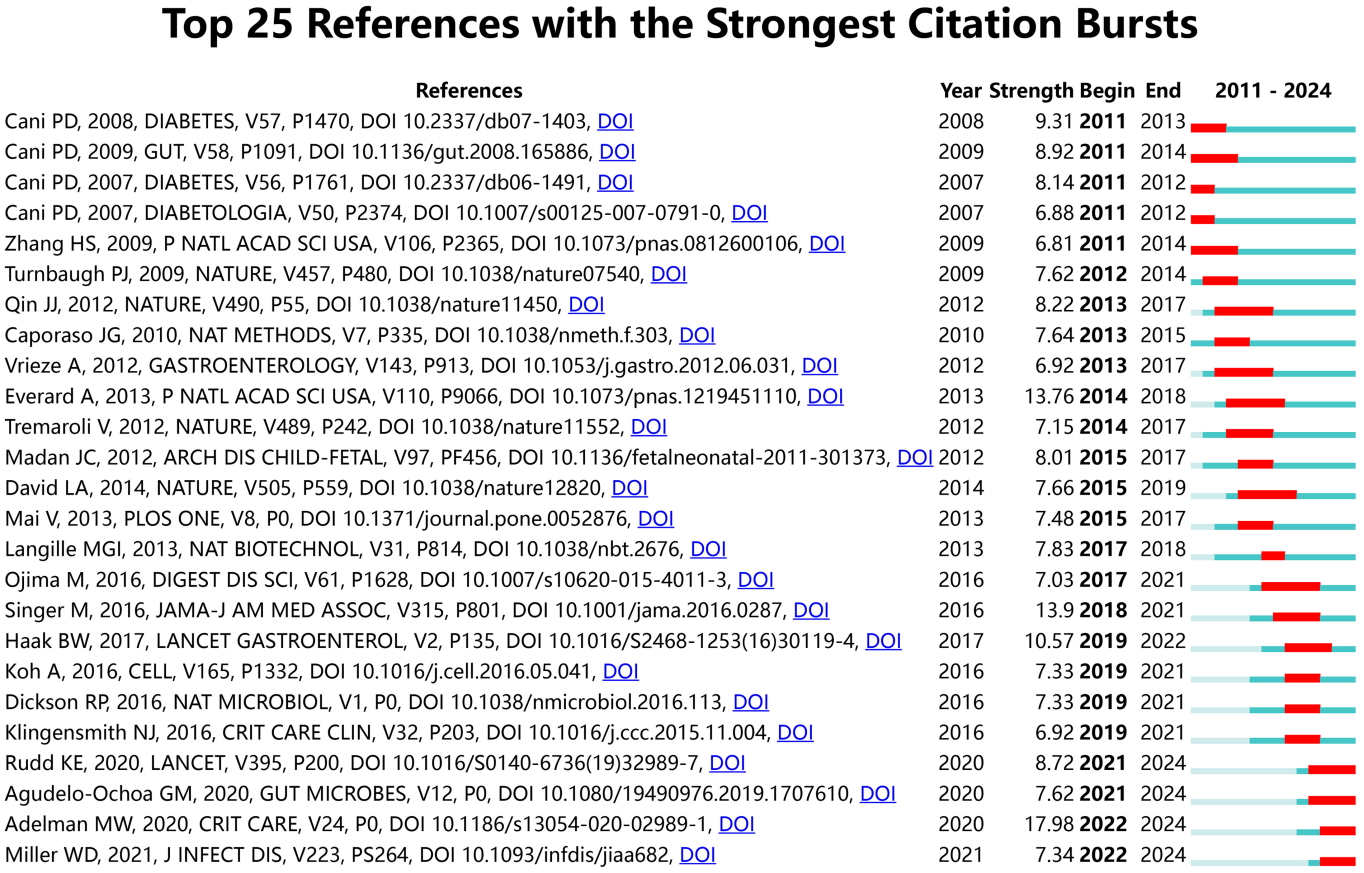
Top 25 References with the Strongest Citation Bursts.

Figure 11 presents a clustered citation network diagram generated using CiteSpace software, identifying 14 relevant clusters: #0 insulin resistance, #1 sepsis-associated acute liver injury, #2 preterm infant, #3 cardiovascular disease, #4 lipopolysaccharide-binding protein, #5 ill patient, #6 emerging therapeutic strategies, #7 gut microbiota, #8 colonic microbiome, #9 proprems trial, #10 necrotizing enterocolitis gut microbe, #11 barrier function mechanism, #12 patient, and #13 model. Using CiteSpace for burst strength analysis of cited references, with a minimum hotspot duration set at 2 years, we identified a total of 189 hotspots, revealing 25 references with significant burst strength. Out of these, 3 references showed burst strengths exceeding 10 (Figure 12). This evaluation emphasizes the most impactful and quickly developing subjects in a particular period, offering a glimpse into the changing trends and primary areas of interest in sepsis and gut microbiota studies.

## 4 Discussion

### 4.1 General information

For this research, we examined 1,031 articles related to sepsis and intestinal microbiota from the WoSCC database utilizing Python, VOSviewer, and CiteSpace. Research shows that since 2021, there has been a substantial annual rise in both the number of publications and citations in this field, largely driven by contributions from China. This demonstrates China’s focus on and significant research contributions in this field. Five out of the top 10 institutions with the highest publication counts are based in China, with Southern Medical University, Zhejiang University, Tongji University in Shanghai, Harbin Medical University, and Zhengzhou University making up this impressive list.

From the analysis of publication volume, the author with the highest output is Leelahavanichkul, Asada from Thailand. However, in the analysis of total citations, Patrice D. Cani from the Catholic University of Louvain in Belgium holds the leading position. Additionally, Nathalie M. Delzenne and Amandine Everard from Belgium, as well as Clara Belzer from the Netherlands, have also been cited over 3,000 times, although they are not among the top 10 in publication count. According to the *H*-index analysis, Leelahavanichkul, Asada (*H*-index of 11) and Cani, Patrice D. (*H*-index of 10) are leading in their respective positions. Antonio Gasbarrini, a researcher at Agostino Gemelli Hospital in Italy, is among the top 10 based on the number of publications but has a relatively low *H*-index of 4. This highlights the importance of considering both publication quantity and quality when assessing academic impact.

From the perspective of journals, *Nutrients* is the most prolific in terms of publication quantity. However, despite only publishing two articles, the total citation count of the *Proceedings of the National Academy of Sciences of the United States of America* is the highest. This journal publishes articles of high quality that have a significant impact.

The most prominent research fields are “Microbiology” and “Immunology,” leading in both publication quantity and total citation count. The field of “Sport Sciences” boasts the highest average citations per paper (ACPP), with only one publication totaling 126 citations. This study, by Motiani et al. (29), published in 2020 in *Medicine & Science in Sports & Exercise*, investigated how two different training modes affect gut metabolism and microbiota. It suggests that exercise training can improve gut microbiota characteristics and reduce endotoxemia, demonstrating the breadth of current research areas and providing inspiration for future research fields. The second highest average citation count is in the field of “Multidisciplinary Sciences,” with 125.33 citations, and it also has the highest total citation count. Multidisciplinary Sciences refers to scientific research that involves multiple disciplines and interdisciplinary collaboration. The significant amount of focus and frequency of references in this area emphasizes the significance of interdisciplinary and multidisciplinary teamwork in furthering research on sepsis and intestinal microbiota.

### 4.2 Research hotspots and frontiers

The fields of sepsis and gut microbiota have garnered increasing attention from scholars worldwide over the years. Based on the bubble chart analysis and citation burst analysis of the author’s keywords, there has been a notable rise in research related to “lipopolysaccharides (LPS),” “short-chain fatty acids (SCFAs),” “probiotics,” “fecal microbiota transplantation (FMT),” and the “gut-liver axis.” This trend not only underscores the growing interest in these topics within the academic community but also suggests that they are expected to serve as central areas of focus and leading paths for further research.

#### 4.2.1 Lipopolysaccharides

Lipopolysaccharide (LPS) is a large glycolipid molecule made up of lipid A (also known as endotoxin), a non-repeating core oligosaccharide, and a distant polysaccharide region (O antigen). It is a crucial element of the outer membrane in gram-negative bacteria (30) and serves a central role in sepsis (31). LPS is a clearly defined pathogen-associated molecular pattern (PAMP) that serves as an early indicator of bacterial infection. Even minimal quantities of LPS discharged by an invading pathogen can trigger a strong innate immune reaction in the host, thereby safeguarding the immune system against additional infection. Lipid A (or endotoxin) is a transmembrane protein associated with the IL1 receptor (32), specifically TLR4 (Toll-like receptor 4) (33). Trace amounts of LPS in host macrophages trigger the activation of TLR4 by lipid A, which in turn stimulates the biosynthesis of multiple inflammatory mediators such as TNF-α and IL1-β (34, 35), and activates the production of costimulatory molecules necessary for adaptive immune responses (32). In monocytes and endothelial cells, lipid A also induces the production of tissue factor (36, 37). Perivascular cells and epithelial cells that line the surfaces of organs and the body contain tissue factor, creating a hemostatic barrier. This barrier offers extra shield for crucial organs like the brain, lungs, and heart (38). These physiological responses are beneficial for local or early bacterial infections and are synergistic. If the LPS response is not appropriately regulated, it can cause an overabundance of inflammation and disruptions in microcirculation, ultimately leading to the development of severe septic shock syndrome that can be fatal. When large amounts of LPS enter the cytosol, they act on intracellular receptors. When combined with LPS, caspases in the cells of the host form oligomers and trigger the activation of various cytotoxic agents, such as gasdermin D (GSDMD), caspase-1, and the purinergic receptor P2X7 (39). Effectors activation initiates the production of IL-1, IL-6, and IL-18 by cells, leading to pyroptosis—an inflammatory form of cell death observed in macrophages, endothelial cells, and epithelial cells (40, 41). These inflammatory changes collectively increase the disruption of the pulmonary endothelial barrier, ultimately leading to sepsis and elevated mortality (42). Therefore, LPS is the most commonly used toxin to simulate sepsis-related acute inflammatory responses. It has garnered increasing attention in the study of sepsis treatment strategies and could potentially serve as a new therapeutic focus for sepsis in the future.

At present, research on novel therapeutic strategies for sepsis involving LPS primarily focuses on three directions. First, *in-situ* neutralization of LPS is widely regarded as a potential intervention to fundamentally eliminate or mitigate the inflammatory response induced by LPS. In animal models, neutralization of monomeric LPS and outer membrane vesicles (OMVs) can block LPS activation, blocking its binding to both extracellular and intracellular receptors, thereby reducing inflammation and restoring autophagy (43, 44). Second, Toll-like receptor-4 (TLR4) and CD14 antagonists are being explored as therapeutic drugs for sepsis. TLR4 is especially adept at identifying endotoxin, leading to the activation of cellular and molecular inflammatory reactions. In animal sepsis models, multiple molecules such as TAK-242, eritoran, and TIRAP decoy peptides block TLR4 signaling at different stages through various modes of action, thereby enhancing the chances of survival for septic mice and lowering cytokine levels (45). Activation of myeloid cells and the subsequent release of pro-inflammatory mediators are initiated by the interaction of bacterial cell wall components, CD14, and co-receptors. Studies have shown that using IC14 (a recombinant anti-CD14 monoclonal antibody) can decrease the response to lipopolysaccharide in models of endotoxemia in both animals and humans (46). However, studies have confirmed that synthetic compounds or natural TLR4 antagonists have failed to pass clinical trials, showing no significant improvement in patient survival rates (47–49). CD14 antagonists are still in phase I clinical trials (46), and more anti-CD14 antibodies as therapeutic agents are still being explored (50). Research is currently being conducted on inhibitors of the caspase family. It has been shown through studies that the caspase family plays a crucial role in triggering and advancing the process of apoptosis (51), and is elevated in lymphocytes of individuals with sepsis. Caspases are thought to promote lymphocyte death (52, 53), making them important targets for the development of anti-apoptotic drugs. Lysophosphatidylcholine, a component of lipoproteins, can inhibit caspase-11 activation (39). In animal research, it has been demonstrated that stearoyl lysophosphatidylcholine has protective properties in preventing sepsis in mice that were induced with intraperitoneal injection of LPS (54). Pep19-2.5 is a peptide that acts as an anti-endotoxin, capable of averting sepsis caused by endotoxemia in living organisms. It also hinders the activation of caspase-11, the secretion of IL-1, and the pyroptotic demise of cells in human monocytes and macrophages when tested in a laboratory setting (55). Nevertheless, caspase inhibitors remain at the animal experimental model stage, and there have been no clinical trials conducted on the use of caspase inhibition therapy in sepsis patients (39). Sepsis is a complex disease that requires the continuous development of diagnostic and therapeutic strategies. The intracellular LPS pathway presents numerous targets. Therefore, combining sepsis treatment with internal LPS receptor antagonists and neutralizing circulating LPS could potentially enhance effectiveness. The application and development of LPS in sepsis research are expected to be further advanced.

#### 4.2.2 Short-chain fatty acids

The gut microbiota is a key element of the bacterial community present in all mammals, serving an essential function in the shaping, operation, and control of the immune system starting from birth (56). Short-chain fatty acids (SCFAs) are among the most common microbial metabolites present in the intestines. They reduce the inflammatory response by decreasing the production of pro-inflammatory substances and increasing the production of anti-inflammatory substances. As an example, propionate and butyrate reduce cellular inflammation by suppressing the production of interleukin 6 (IL-6) and reactive oxygen species (ROS), and simultaneously boosting the production of IL-10 (57). Acetate aids in reducing neutrophil inflammation by inducing caspase-dependent apoptosis of neutrophils, reducing the activity of nuclear factor-kappa B (NF-κB), and enhancing the production of anti-inflammatory mediators such as IL-10, transforming growth factor-beta (TGF-β), and annexin A1 (58). In a study using LPS-treated human pulmonary microvascular endothelial cells (HPMECs), it was found that sodium propionate not only facilitated the translocation of Nrf2 into the nucleus, safeguarded the cells, and enhanced angiogenesis, but also decreased the inflammatory reaction via the NF-B pathway (59). Short-chain fatty acids (SCFAs) also play a role in controlling the activity of innate immune cells and can impact the development and function of T-cells and B-cells, which in turn affects antigen-specific adaptive immunity (60). Research has verified that short-chain fatty acids stimulate the production of IL-22 by CD4 T cells by binding to the receptor G-protein-coupled receptor 41 (GPR41) and inhibiting histone deacetylase (HDAC) (61). Short-chain fatty acids (SCFAs) enhance B-cell differentiation by boosting acetyl-CoA levels, glycolysis, fatty acid synthesis and oxidative phosphorylation (62). Nevertheless, not all research has supported the anti-inflammatory and immunomodulatory effects of SCFAs. It is possible for SCFAs to be ineffective or even come with side effects. Some studies have revealed that SCFAs stimulate FFA2 and FFA3 receptors in neutrophils and macrophages. The pro-inflammatory outcomes of activating FFA2 and FFA3 receptors are associated with the activation of MAPK, PI3K, or mTOR signaling pathways (63). Additionally, SCFAs can increase the production of cytokines (IL-6, CXCL1, and CXCL2) by activating the extracellular signal-regulated kinase 1/2 (ERK1/2) and p38MAPK signaling pathways (64). When elevated levels of SCFAs attach to particular TLR ligands, they have the ability to increase the release of pro-inflammatory cytokines and stimulate the generation of pro-inflammatory cytokines (65). Hence, due to their dual impact of both pro-inflammatory and anti-inflammatory activities, the potential therapeutic benefits of SCFAs in treating and controlling diseases, particularly those related to immune responses, warrant further investigation.

Sepsis-related encephalopathy (SAE) is a prevalent form of brain dysfunction in patients with sepsis. The homeostasis of the “microbiota-gut-brain axis” in these patients is disrupted, leading to gut microbiota disturbances and a reduction in the concentration of various SCFA components in feces and blood. The reduction in SCFAs concentration leads to cognitive decline. This is associated with an increase in GFAP-positive cells in the prefrontal cortex and hippocampus (66–68). Research has shown that short-chain fatty acids (SCFAs) can prompt naive CD4 T-cells to develop into Treg cells, offering a viable option for managing autoimmune conditions (69, 70). Li et al. (71) suggested that short-chain fatty acids (SCFAs) could potentially enhance hippocampal neuroinflammation by stimulating the colonic NLRP6 inflammasome independently of peroxisome proliferator-activated receptor (PPAR-) activation. This process could also lead to increased levels of DCX-positive new neurons in the hippocampus. Deitch (72) proposed that gut microbiota and certain metabolites may travel from the intestines to distant organs by way of the portal vein or pass through the thoracic duct via mesenteric lymph nodes. This process could lead to them entering the bloodstream and influencing the brain. Hoyles et al. (73) showed that propionate, a short-chain fatty acid (SCFA), had a protective impact against oxidative stress on the blood-brain barrier (BBB) by activating the nuclear factor erythroid 2-related factor 2 (NRF2, also refer blue to as Nfe2l2) signaling pathway. Therefore, it is indeed feasible to use SCFA treatment to maintain SCFA concentration after the onset of sepsis and as a dietary intervention for SAE. Further research is needed to establish both the qualitative and quantitative standards for SCFA species and abundance. Moreover, it is essential to investigate the intricate interactions of SCFAs in the human body, particularly within the framework of the “microbiota-gut-brain axis,” and to delve deeper into how SCFAs impact gene expression in brain cells.

#### 4.2.3 Probiotics

Probiotics, which are beneficial intestinal microorganisms, perform several crucial functions, including immune regulation, pathogen prevention, improvement of intestinal barrier function, and specifically encouraging the proliferation and function of a small group of bacteria in the gut (74, 75). These functions have the potential to lower the likelihood of sepsis and enhance sepsis results in certain groups of patients (76, 77). Probiotics regulate inflammatory pathways in epithelial and immune cells and influence gene expression within the immune system, including the activation of IL-6, MAPKs, IL-8, B-cell-protein-kinase (NF-κB) and nuclear factors of TNF-α (78). Multiple randomized controlled trials have shown that probiotic treatment effectively enhanced the diversity of fecal bacteria in early sepsis patients (79). The administration of synbiotics containing *Lactobacillus plantarum* has been associated with a significant reduction in neonatal sepsis and mortality (77). Probiotics can effectively reduce the proportion of NKT cells and the levels of inflammatory factors in septic children, and regulating the intestinal tract can play a role in protecting lung function (80), which is of positive significance in improving the long-term prognosis of septic children. Nevertheless, the simultaneous administration of broad-spectrum antibiotics to sepsis patients might impede the colonization and positive impacts of probiotics (81). Additionally, the effects of probiotics may be specific to the studied formula, and differences in formulas can reduce the overall signal of probiotic efficacy (82, 83). Hence, further mechanistic research is required to identify specific next-generation probiotic strains and to explore various combinations in order to gain a deeper understanding of how probiotics mechanistically impact sepsis.

#### 4.2.4 Fecal microbiota transplantation

Fecal microbiota transplantation (FMT) involves transferring the feces of healthy donors, that house thousands of bacterial colonies, to the intestines of patients for recolonization after minimal treatment. In critically ill patients, more than half of the commensal microbiota is lost within hours of injury, leading to the rapid overgrowth of potentially pathogenic and pro-inflammatory bacteria. This change affects metabolism, immunity, and even neurocognitive functions, making the intestine the cause of systemic inflammation and multi-organ failure (84–86). In the intensive care unit, restoring a healthy microbiota through FMT is both reasonable and effective. FMT operates by altering the expression of IRF3 and enhancing the presence of butyrate-producing bacteria can alter the systemic immune response to infection. This restoration of IRF3 expression aids in the clearance of pathogenic pathogens in response to sepsis (87).

However, the widespread use of FMT is primarily limited by the need to discontinue antibiotics (88). Antibiotics are generally considered a key component of the treatment regimen for sepsis, making it difficult to reach a consensus on FMT in sepsis treatment. At present, there is no effective way to detect potential harmful bacteria in donor samples. Individuals with severe sepsis, systemic inflammatory response syndrome, and multiple organ dysfunction may encounter significant and possibly life-threatening complications during fecal microbiota transplantation. Therefore, in the future, targeted FMT therapy or the delivery of specific bacterial communities that can restore the function of specific microbiota could provide a more controllable approach in the treatment of sepsis with FMT.

### 4.3 Gut-liver axis

The gut-liver axis, also known as gut-liver crosstalk, is a rapidly growing area of study that focuses on the two-way connection between the gut and its microbiota and the liver. This barrier restricts the passage of microorganisms and toxins throughout the system, but permits nutrients to pass through and reach the liver (89). In sepsis, a compromised gut barrier and disruption of the gut microbiota result in the transmission of pathogen-associated molecular patterns (PAMPs) and damage-associated molecular patterns (DAMPs) from the intestines to the liver and throughout the body. This transfer triggers a pro-inflammatory cascade, exacerbating liver inflammation (90, 91). Liver dysfunction, decreased bacterial clearance rate, and metabolic disorders further deteriorate intestinal function, leading to coagulation dysfunction, endocrine disorders, metabolic disturbances, and ultimately multiple organ failure (MOF) (92). Inflammation significantly exacerbates sepsis-induced intestinal injury and alters intestinal permeability (93, 94). The impairment of the intestinal barrier results in the failure of the defensive luminal mechanism, allowing a large amount of lipopolysaccharides (LPS) to enter systemic circulation (95). LPS, along with intestinal-derived PAMPs and DAMPs, migrates to multiple organs outside the intestine, triggering uncontrolled immune inflammatory responses, impaired clearance of liver pathogenic bacteria, and metabolic disorders (96–98). New therapeutic targets focusing on the gut-liver axis are under development, including epithelial barrier-targeted therapy, targeting the gut microbiome, duodenal mucosal resurfacing, intestinal restrictive polymers, and intestinal peptides. In-depth study of the gut-liver axis not only advances the management, diagnosis, and treatment of liver disease but also helps prevent and limit liver injury caused by sepsis, thereby improving the prognosis of patients with sepsis.

In patients with sepsis, a normal liver can engulf invading pathogenic microorganisms and their metabolites to participate in the immune inflammatory response, aiding the body in regulating immune defense. Conversely, impaired liver detoxification further aggravates the development of sepsis. Liver dysfunction often indicates critical illness and poor prognosis, serving as an independent predictor of sepsis outcomes. Hence, timely prevention and treatment of liver damage are essential for enhancing the outcomes of patients with sepsis. However, the specific mechanisms of gut-liver axis disorder in the pathological progression of sepsis remain largely mysterious and require further exploration and research.

In conclusion, this research comprehensively examined the intricate relationships between sepsis and the gut microbiota, identifying five key author keywords that represent areas of significant research interest and future exploration. Specifically, this paper discusses LPS, SCFAs, probiotics, FMT, and the gut-liver axis in the context of intestinal flora and sepsis. Each keyword highlights the progress of current research and identifies directions for future exploration.

### 4.4 Strengths and limitations

In this study, bibliometric methods were employed to visually analyze the relationship between sepsis and intestinal flora, presenting a thorough analysis of the advancement patterns in this area of research for the first instance. This method provides a structured guide for academics and enhances our comprehension of the present circumstances, focal points, and developments in this field. Additionally, the Python code used in this study can automatically read the txt file of literature citation information and quickly generate a bubble chart to present the research results.

However, our study also has limitations. The Web of Science Core Collection (WoSCC) database is highly respected as a reliable source in scientific publishing. Nonetheless, it does not include articles from non-SCI journals or other databases such as PubMed, Cochrane Library and Google Scholar, potentially resulting in exclusion of certain studies. Additionally, the bibliometric method heavily depends on citation indicators, which do not provide a comprehensive assessment of the internal quality of individual studies. In addition, the frequency of citations is affected by the passage of time, with newer studies typically receiving fewer citations because they were recently published. In conclusion, there could exist notable discrepancies in the categorization of subject headings and the application of keywords in different bodies of literature, which may lead to bias and fail to accurately represent the real scenario. Although these restrictions could impact the findings, they are not likely to change the fundamental patterns uncovered in this research.

## 5 Conclusion

In this research, bibliometric techniques are used in conjunction with Python programming language, VOSviewer, and CiteSpace software to conduct a thorough analysis of the scholarly works on sepsis and intestinal flora that were released in the WoSCC database between 2011 and 2024. According to the findings, this field has consistently remained a key focus of scientific research. China is notable on an international level for excelling in the number of published papers, the influence of research institutions, and the caliber of esteemed scholars, placing the United States in second position. By conducting a thorough examination of author keywords and citation burst analysis, this research pinpointed LPS, SCFAs, probiotics, FMT, and the gut-liver axis as key areas of focus and possible future pathways within the subject area. These topics not only highlight the complex relationship between sepsis and intestinal flora but also suggest potential directions for future research. As this field continues to evolve, interdisciplinary research will become increasingly important. This study aids new scholars in obtaining a clearer and quicker understanding of the global research status in the field of sepsis and intestinal flora. Furthermore, it offers essential reference materials for organizations or parties interested in collaborating on scientific research in this field.

## Data Availability

The original contributions presented in the study are included in the article/supplementary material, further inquiries can be directed to the corresponding author.
